# Germacrene B – a central intermediate in sesquiterpene biosynthesis

**DOI:** 10.3762/bjoc.19.18

**Published:** 2023-02-20

**Authors:** Houchao Xu, Jeroen S Dickschat

**Affiliations:** 1 Kekulé-Institute of Organic Chemistry and Biochemistry, University of Bonn, Gerhard-Domagk-Straße 1, 53121 Bonn, Germany

**Keywords:** biosynthesis, configuration determination, germacrene B, structure elucidation, terpenes

## Abstract

Germacranes are important intermediates in the biosynthesis of eudesmane and guaiane sesquiterpenes. After their initial formation from farnesyl diphosphate, these neutral intermediates can become reprotonated for a second cyclisation to reach the bicyclic eudesmane and guaiane skeletons. This review summarises the accumulated knowledge on eudesmane and guaiane sesquiterpene hydrocarbons and alcohols that potentially arise from the achiral sesquiterpene hydrocarbon germacrene B. Not only compounds isolated from natural sources, but also synthetic compounds are dicussed, with the aim to give a rationale for the structural assignment for each compound. A total number of 64 compounds is presented, with 131 cited references.

## Introduction

Terpenoids constitute the largest class of natural products with ca. 100,000 known compounds. Biosynthetically, all terpenoids are derived from only a few acyclic precursors, including the monoterpene precursor geranyl diphosphate (GPP) [1], the precursor for sesquiterpenes farnesyl diphosphate (FPP) [2], geranylgeranyl diphosphate (GGPP) towards diterpenes [3], and the sesterterpene precursor geranylfarnesyl diphosphate (GFPP) [4]. It has been demonstrated recently, that even farnesylfarnesyl diphosphate (FFPP) can serve as a precursor to triterpenes [5], a compound class that was believed to be solely derived from squalene. Terpene synthases convert these linear precursors through cationic cascade reactions into terpene hydrocarbons or alcohols [6–8]. For type I terpene synthases this multistep process is initiated by the abstraction of diphosphate to produce an allyl cation that subsequently undergoes typical cation reactions such as cyclisations by intramolecular attack of an olefin to the cationic centre, Wagner–Meerwein rearrangements, hydride or proton shifts. The process is terminated by deprotonation to yield a terpene hydrocarbon or by nucleophilic attack of water to generate a terpene alcohol.

**Scheme 1:**
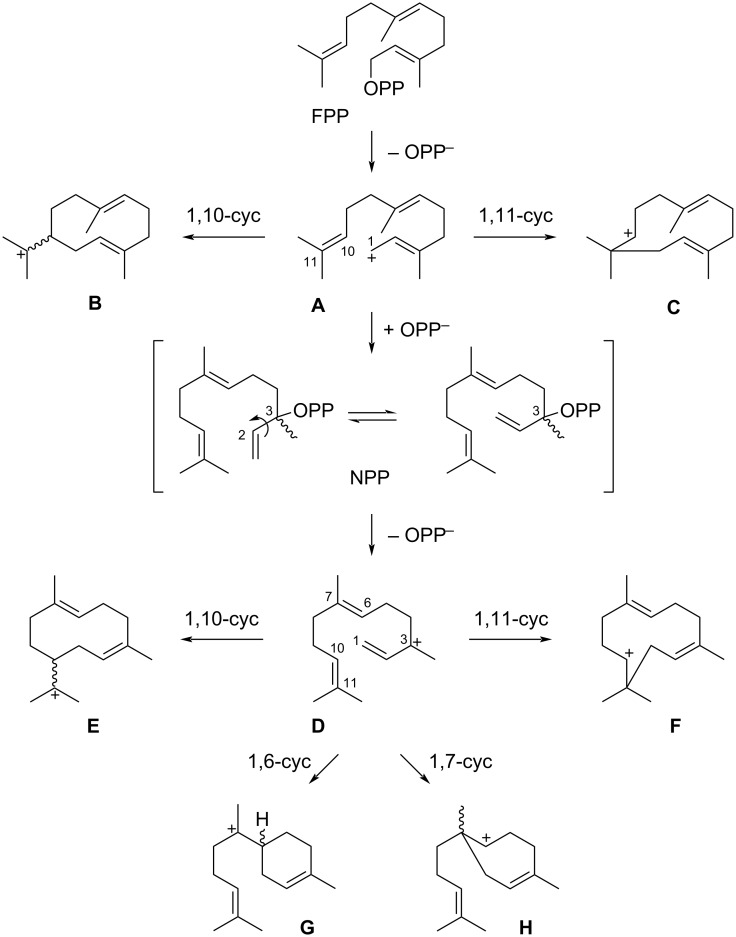
Possible cyclisation modes of FPP.

For the precursor of sesquiterpenes FPP six initial cyclisation modes are possible (Scheme 1). After ionisation to **A** either a 1,10-cyclisation to the (*E*,*E*)-germacradienyl cation (**B**) or a 1,11-cyclisation to the (*E*,*E*)-humulyl cation (**C**) is possible. Reattack of diphosphate at C-3 results in nerolidyl diphosphate (NPP) that can undergo a conformational change by rotation around the C-2/C-3 single bond, which allows reionisation to **D**. This intermediate can react in a 1,10-cyclisation to the (*Z*,*E*)-germacradienyl cation (**E**) or a 1,11-cyclisation to the (*Z*,*E*)-humulyl cation (**F**), the *E*/*Z* stereoisomers of **B** and **C**. Furthermore, a 1,6-cyclisation to the bisabolyl cation (**G**) or a 1,7-cyclisation to **H** may follow, which is not possible from **A** because of its 2*E* configuration (a hypothetical (*E*)-cyclohexene or (*E*)-cycloheptene would be too strained, the smallest possible ring with an *E* configuration is (*E*)-cyclooctene).

**Scheme 2:**
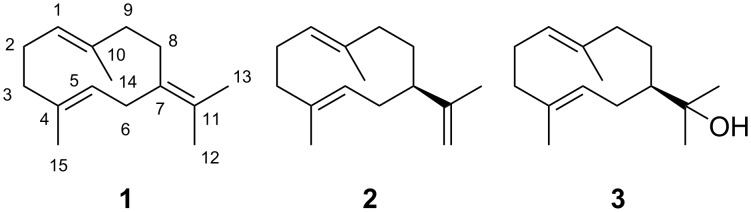
Structures of germacrene B (**1**), germacrene A (**2**) and hedycaryol (**3**).

In some cases the initially formed neutral product can become reprotonated to initiate a second round of cyclisation reactions which usually leads to compounds of higher structural complexity. It was already noticed in the 1950s by Ruzicka [9] and Barton and de Mayo [10], followed by a more detailed elaboration by Hendrickson [11], that 10-membered sesquiterpenes such as hedycaryol (**3**) can serve as neutral intermediates that can react upon reprotonation to 6-6- (selinane) or 5-7-bicyclic (guaiane) sesquiterpenes. We have recently summarised the accumulated knowledge about sesquiterpenes derived from germacrene A (**2**) [12] and hedycaryol (**3**) [13]. Now we wish to provide a review on the known chemical space of sesquiterpenes derived from germacrene B (**1**) (Scheme 2). Compounds derived from **1** by oxidation will not be included in this article. The interested reader can find exemplary relevant information about this topic in references [14–18].

## Review

### Germacrene B

**Scheme 3:**
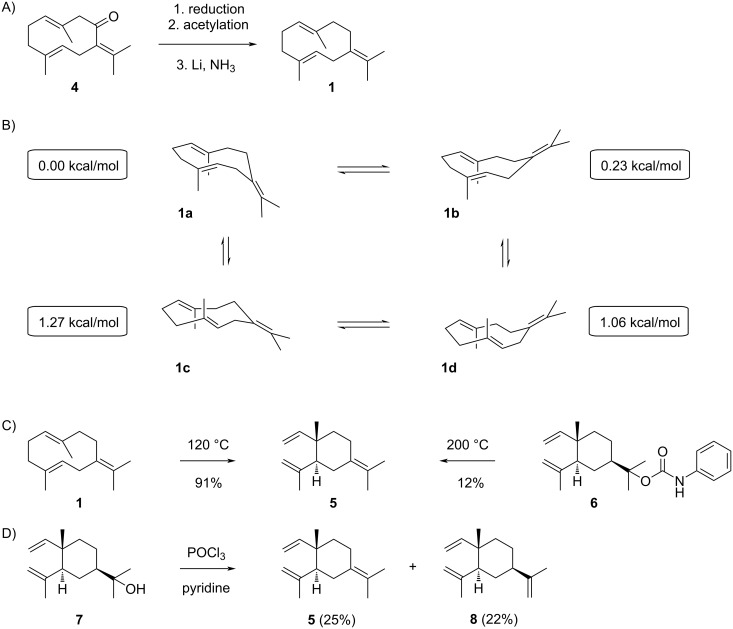
The chemistry of germacrene B (**1**). A) Synthesis from germacrone (**4**), B) the four conformers of **1** established by molecular mechanics calculations (energies in black boxes are relative to **1a** for which the energy was set to 0.00 kcal/mol), C) Cope rearrangement to **5** and formation from **6** by pyrolysis, D) dehydration of **7** to **5** and **8**.

Germacrene B (**1**) was first prepared from germacrone (**4**), a compound identified by Šorm and co-workers [19], through a sequence of reduction to the alcohol, acetylation and reduction with lithium in ammonia (Scheme 3A) [20], and its structure was unambiguously assigned by X-ray crystallography of a silver nitrate adduct [21]. From natural sources, the compound was first obtained from *Humulus lupulus* by preparative gas chromatography [22] and from *Citrus junos* [23], followed by isolations from *Stenocalyx michelii* [24], *Citrus aurantifolia* [25], and *Solidago canadensis* [26]. Germacrene B has been ascribed a warm, sweet, woody-spicy, geranium-like odour and is an important flavour constituent of lime peel oil [25]. Germacrene B is also one of the main constituents of the essential oils from different plants that have antibacterial activity [27–29]. Germacrene B synthases have been reported from *Solanum habrochaites* [30] and *Cannabis sativa* [31]. In addition, **1** is a minor product of the germacrene C synthase from *Lycopersicon esculentum* [32], the (+)-germacrene D synthase from *Zingiber officinalis* (17.1%) [33], the avermitilol synthase from *Streptomyces avermitilis* (5%) [34], and VoTPS1 from *Valeriana officinalis* [35]. For the bacterial selinadiene synthase (SdS) from *Streptomyces pristinaespiralis* **1** is an intermediate in the cyclisation of farnesyl diphosphate (FPP) to selina-4(15)-7(11)-diene [36]. Several SdS enzyme variants have been constructed by site-directed mutagenesis, including the enzyme variants D83E, E159D and W304L, for which the product spectrum is shifted towards **1** as the main product [36].

Based on molecular mechanics calculations, four conformers **1a**–**d** have been described for **1** (Scheme 3B) [37]. The calculations revealed all four conformers are of similar stability, with **1a** being the most stable conformer. The fact that **1** shows a defined set of fifteen sharp signals in the ^13^C NMR spectrum [26] indicates that the interconversion between these conformers is a fast process at room temperature. This is in contrast to the findings for germacrene A (**2**) and hedycaryol (**3**) that show strong line broadening in the NMR spectra and multiple sets of peaks for different conformers [26,38–41], pointing to a higher energy barrier between their conformers in comparison to the barriers between the conformers of **1**. Like observed for germacrene A [40] and hedycaryol [41–42], **1** readily undergoes a Cope rearrangement to γ-elemene (**5**) above 120 °C (Scheme 3C), while the reaction of **1** with bis(benzonitrile) palladium chloride generates the palladium chloride complex of **5** from which **5** can be liberated by treatment with dimethyl sulfoxide [43]. Compound **5**, with tentatively assigned structure, was first obtained as a pyrolysis product of elemol phenylurethane (**6**) [44]. Its structure was subsequently secured by preparation from **1** through Cope rearrangement [20] and through dehydration of elemol (**7**) with POCl_3_ in pyridine yielding **5** and β-elemene (**8**) (Scheme 3D) [45]. Compound **5** has also frequently been reported from natural sources especially after heat treatment of the sample, and has been isolated from *Cryptotaenia japonica* [46], *Bunium cylindricum* [47], an unidentified *Pilocarpus* sp. [48], and *Aristolochia triangularis* [49].

**Scheme 4:**
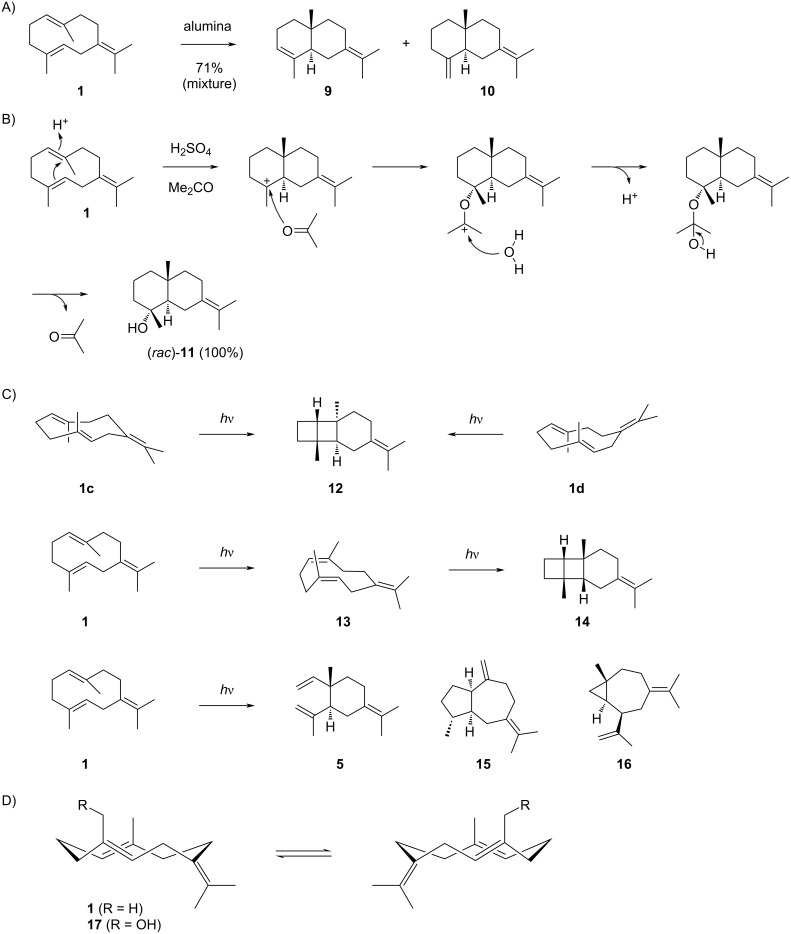
The chemistry of germacrene B (**1**). A) Cyclisation of **1** to **9** and **10** upon treatment with alumina, B) conversion into (*rac*)-**11** by treatment with diluted sulfuric acid in acetone, C) photochemical products from **1**, and D) planar chirality of **1** and its derivative **17**.

Germacrene B (**1**) is also easily cyclised to selinanes. Percolation of **1** through alumina yields a 1:1 mixture of selina-3,7(11)-diene (**9**) and γ-selinene (**10**) (Scheme 4A) [43]. Interestingly, while racemic juniper camphor (**11**) is formed from **1** upon acid treatment [50], this reaction with diluted sulfuric acid in acetone results in (*rac*)-**11** quantitatively. This observation is explained by a protonation-induced cyclisation, successive addition of acetone and water to a hemiacetal that can decompose to **11** (Scheme 4B) [43]. Furthermore, **1** shows an interesting photochemistry (Scheme 4C). A [2 + 2] cycloaddition of the endocyclic double bonds yields **12** whose formation is understandable from conformers **1c** and **1d**. The all-*cis* stereoisomer **14** requires a photochemical *E*/*Z* isomerisation to **13** prior to [2 + 2] cycloaddition. Further photochemical products from **1** include **5**, **15** that may be formed through a biradical mechanism, and rearranged **16** [51]. Germacrene B (**1**) has planar chirality (Scheme 4D), but recovery of the starting material from an incomplete Sharpless epoxidation of its derivative 15-hydroxygermacrene (**17**) showed that this material was racemic, indicating a rapid interconversion between the enantiomers of **17**. Consequently, also the enantiomers of **1** may undergo a fast interconversion [52]. The ^1^H and ^13^C NMR data of **1** have been reported [26].

**Scheme 5:**
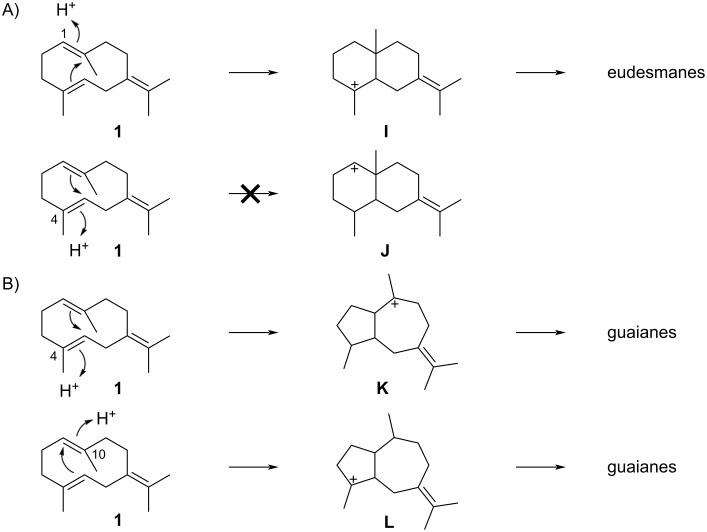
Possible cyclisation reactions upon reprotonation of **1**. A) Cyclisations to eudesmane sesquiterpenes, B) cyclisations to guaiane sesquiterpenes.

Upon reprotonation germacrene B (**1**) can in theory yield several cyclisation products with distinct skeletons. Eudesmanes can be obtained through reprotonation at C-1 and cyclisation to intermediate **I**, or through reprotonation at C-4 leading to cation **J** (Scheme 5A). Further cyclisation modes include a reprotonation at C-4 and cyclisation to **K** or reprotonation at C-10 and cyclisation to **L**, which represent possible precursors of guaianes (Scheme 5B). For all four intermediates **I**–**L** different stereochemistries may be realised. In principle, these reactions may be enzyme catalysed or proceed without enzyme catalysis, e.g., during chromatographic purifications of compounds from complex extracts. In the latter case, because of the achiral nature of **1**, racemic mixtures are expected, while enzyme products should usually be enantiomerically pure or enriched.

### Eudesmanes

**Scheme 6:**
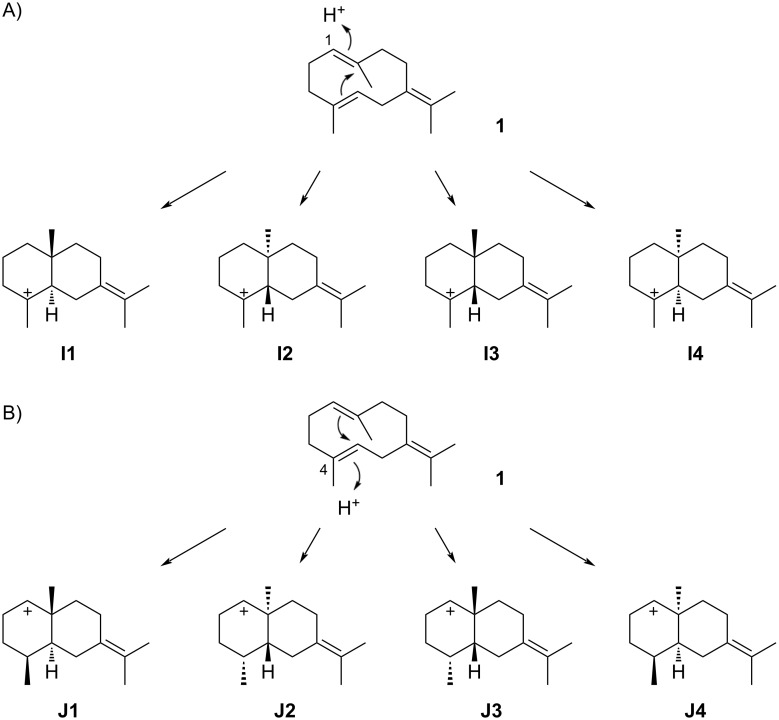
Cyclisation modes for **1** to the eudesmane skeleton. A) The reprotonation of **1** at C-1 potentially leads to four stereoisomers of cation **I**, B) reprotonation at C-4 potentially leads to four stereoisomers of **J**.

The eudesmane skeleton can arise by reprotonation at C-1 of **1**, leading to four different stereoisomers of cation **I**, i.e., **I1** with a *trans*-decalin skeleton, its enantiomer **I2**, **I3** representing the *cis*-decalin skeleton, and its enantiomer **I4** (Scheme 6A). In principle, the eudesmane skeleton can also be formed through cyclisations induced by reprotonation at C-4. Assuming *anti* addition to the C-4/C-5 double bond, these reactions lead to four stereoisomers of the secondary cation **J**, two with a *trans*-decalin skeleton (**J1** and **J2**) and two with a *cis*-decalin skeleton (**J3** and **J4**). However, no natural products are known that may arise through any of these cations **J**, showing that a cyclisation of **1** induced by reprotonation at C-4 is not preferred. Also no compounds have been isolated with their structures rigorously elucidated that arise through cation **I4**. For compounds potentially generated through intermediates **I1**–**I3** the accummulated knowledge will be discussed in the following sections.

### Eudesmanes from **I1**

**Scheme 7:**
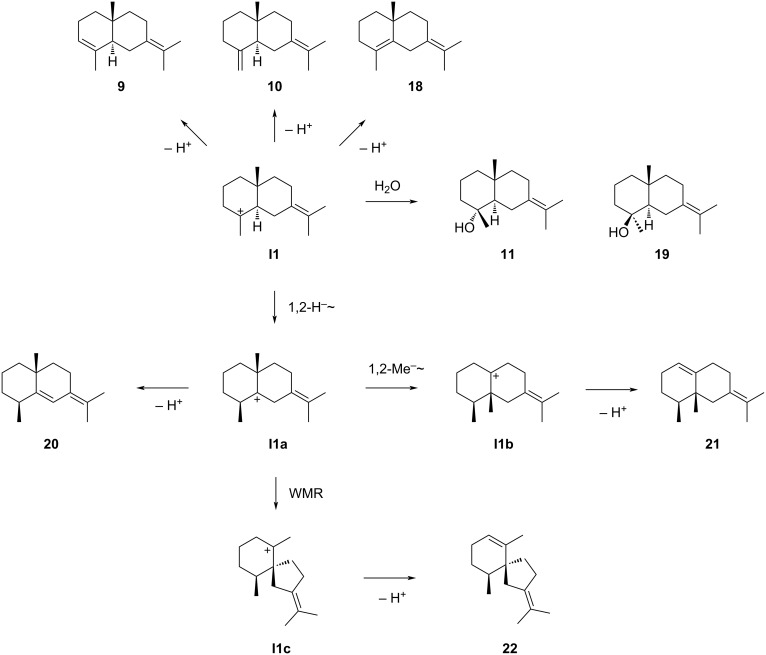
The sesquiterpenes derived from cation **I1**. WMR = Wagner–Meerwein rearrangement.

The eudesmane sesquiterpenes derived from cation **I1** are summarised in Scheme 7. Cation **I1** can either be deprotonated to yield selina-3,7(11)-diene (**9**), (+)-γ-selinene (**10**) or (+)-selina-4,7(11)-diene (**18**), or captured by water resulting in juniper camphor (**11**) or 4-*epi*-juniper camphor (**19**). γ-Selinene (**10**) was first obtained by Šorm and co-workers from wormwood oil (*Artemisia absinthum*). Its positive optical rotation ([α]_D_^25^ = +2.8) [53] suggests an enzymatic formation from **1** in this species. Compound **9**, along with **10**, was first isolated from *Humulus lupulus*, and the structures of both compounds were elucidated by ^1^H NMR spectroscopy and catalytic hydrogenation, yielding the same compound selinane in both cases [54]. Both compounds were later also isolated from *Cannabis sativa* [55]. Unfortunately, no optical rotations were given in these reports, so it remains unknown if the isolated materials arose from **1** by enzymatic or acid-catalysed reactions.

**Scheme 8:**
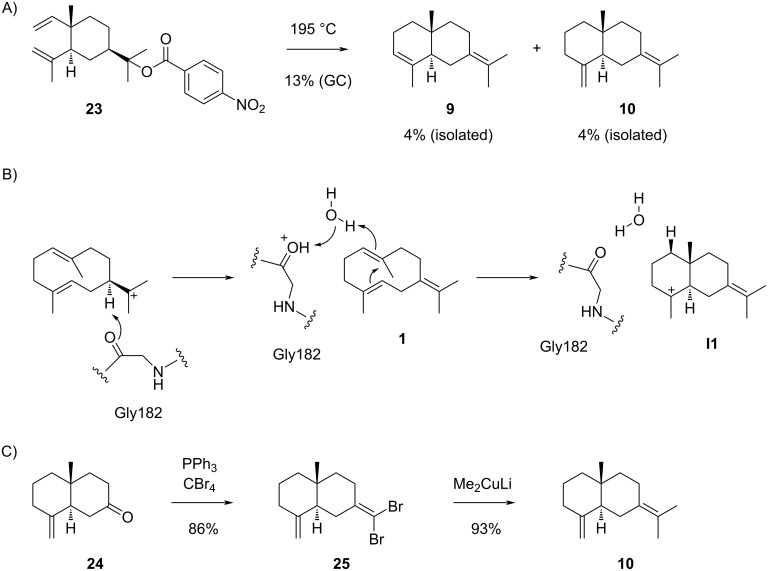
The sesquiterpenes derived from cation **I1**. A) Pyrolysis of **23** to yield **9** and **10**, B) deprotonation–reprotonation sequence in the biosynthesis of **10** by selinadiene synthase, C) synthesis of **10** from **24**.

The sesquiterpenes **9** and **10**, besides several other products, were also prepared through pyrolysis of elemyl *p*-nitrobenzoate (**23**) (Scheme 8A) [56]. Because of the enantiomerically pure starting material, the products were obtained in enantiomerically pure form, showing an optical rotation of [α]_D_ = −6.0 (*c* 0.484) for **10**, while no data were given for the optical rotation of **9**. Compound **9** was also isolated from *Asarum caulescens* ([α]_D_^25^ = −5.5, *c* 0.4, MeOH) [57]. Despite the opposite sign for the optical rotation as reported by Šorm and co-workers [53], the same absolute configuration of **9** is shown in this report. Furthermore, **9** has been described as a marker of the Lemberger variety of grapes (*Vitis vinifera*) [58]. Additional sources from which **10** has been isolated include *Persea japonica* [59], *Solidago canadensis* [60], *Citrus nobilis* ((+)-form) [61], *Zingiber officinalis* [62], *Myrica pensylvanica* and *M. macfarlanei* [63], *Trichogonia scottmorii* [64], and *Podocarpus spicatus* in which case a high optical rotation was reported ([α]_D_^20^ = +82, *c* 2.9, CHCl_3_) [65].

The sesquiterpene **9** is a side product of the δ-selinene synthase (ag4) from *Abies grandis* [66] and a product of several terpene synthases from *C. sativa* (CsTPS7, CsTPS8 and CsTPS22) [67], while **10** is the main product of the bacterial selinadiene synthase from *Streptomyces pristinaespiralis* [36,68]. It has recently been shown by a combined computational and experimental approach that in this enzyme the main chain carbonyl oxygen of Gly182 near the helix G kink and an active site water are involved in the deprotonation–reprotonation sequence in the biosynthesis of **10** (Scheme 8B) [69]. γ-Selinene (**10**) has been synthesised from ketone **24** through conversion into the dibromoalkene **25** with PPh_3_ and CBr_4_, followed by treatment with Me_2_CuLi (Scheme 8C) [70]. NMR data for **9** [71] and for **10** [59] have been published.

**Scheme 9:**
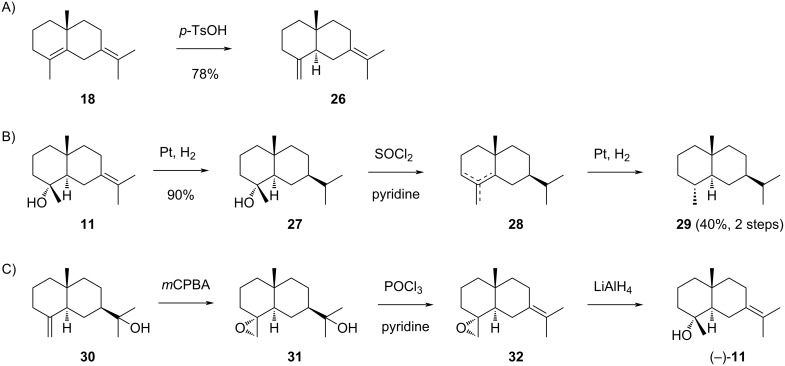
The sesquiterpenes derived from cation **I1**. A) Acid-catalysed conversion of **18** into **26**, B) conversion of **11** into **29** showing that **11** is a selinane sesquiterpene alcohol, C) synthesis of (−)-**11** from **30** (yields were not specified in the original report).

Selina-4,7(11)-diene (**18**), [α]_D_^24^ = +34 (*c* 0.90), was first isolated from the marine alga *Laurencia nidifica*. Its structure was determined by NMR spectroscopy and verified by the acid-catalysed conversion into δ-selinene (**26**) (Scheme 9A) [72]. The same compound **18** was also reported from the closely related alga *Laurencia nipponica* [73] and from lime oil (*Citrus aurantifolia*) [74]. Fully assigned ^1^H and ^13^C NMR data were reported for **18** [72,74].

The structure elucidation of juniper camphor (**11**), a compound originally isolated by chemists at Schimmel, the world leading company of the late 19th and early 20th century dealing with essential oils and perfumes, was initiated by Šorm and co-workers [75]. From the sequence of catalytic hydrogenation to **27**, dehydration to a mixture of alkenes (**28**) and hydrogenation to selinane (**29**) it was concluded that **11** was a selinane sesquiterpene alcohol (Scheme 9B) [75]. Four years later, based on NMR data Bhattacharyya and co-workers suggested a *cis*-ring junction for **11** [76], but a synthesis from β-eudesmol (**30**) through epoxidation to **31**, dehydration to **32** and epoxide opening with LiAlH_4_ yielded (−)-**11** (Scheme 9C) [77], contradicting this assignment.

Notably, Šorm and co-workers noticed that **11** was racemic, because neither **11** nor any of its degradation products showed optical activity [75], suggesting that the compound they had isolated arose through acid-catalysed cyclisation of **1** rather than in an enzymatic process. Also the material isolated from *Platysace linearifolia* showed no optical rotation [78], while the optical activity of **11** isolated from *Bunium cylindricum* [47], *Acritopappus prunifolius* [79], *Aniba riparia* [80], *Juniperus oxycedrus* [81], and *Laggera alata* [82] has not been determined. The (−)-enantiomer of **11** with the structure as shown in Scheme 9C was reported from *Cabralea cangerana* ([α]_D_^20^ = –1.3, *c* 1.3, CDCl_3_) [83], *Zanthoxylum naranjillo* (no value specified) [84], and *Chiloscyphus polyanthos* ([α]_D_ = −3.0, *c* 2.41, CHCl_3_) [85]. The (+)-enantiomer of **11** is known from *Cinnamomum camphora* ([α]_D_^25^ = +1.79), representing the first isolated enantiomerically enriched material [86]. The low value of the optical rotation of **11** makes configurational assignments based on optical activity difficult, especially if minor contaminants falsify these data. Furthermore, the variability of the optical rotations given in the literature may be a consequence of mixed enantiomeric compositions arising from contaminations of enzymatically formed **11** with **11** generated upon acid catalysis during compound isolations. The reporting of (–)-**11**, (+)-**11** and **11** of unspecified absolute configuration all under the same CAS number (473-04-1) adds to the confusion. Moreover, one report is available that mentions the isolation of **11** from *Atractylodes macrocephala* [87]. For unclear reason, this paper is assigned to CAS number 1647153-38-5 representing the structure of **19** (Scheme 7), which actually seems to be an unknown compound.

Compound **11** is a side product of ZmTPS7 from *Zea mays* [88] and ^1^H and ^13^C NMR data for **11** have been published [82–83]. A recent molecular docking study suggested that **11** can bind to the main protease M^pro^ of the SARS-CoV-2 virus that is involved in viral reproduction, but experimental tests supporting this finding are lacking [89].

**Scheme 10:**
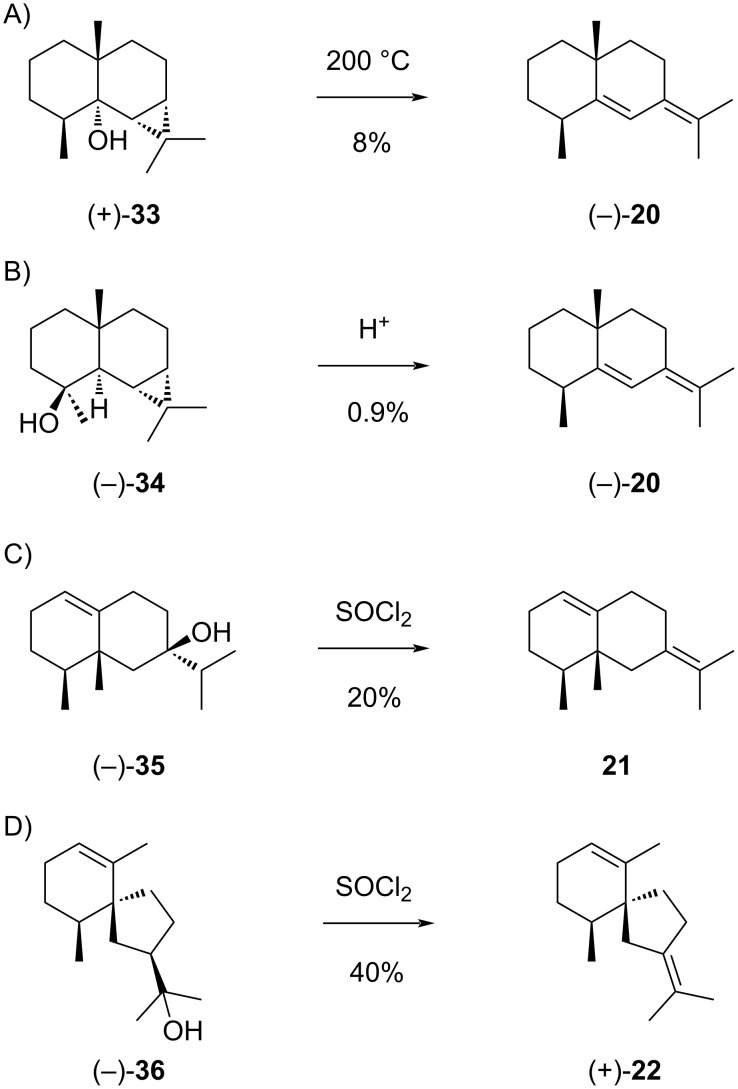
The sesquiterpenes derived from cation **I1**. A) Formation of **20** by pyrolysis of **33**, B) acid-catalysed dehydration of **34** to **20**, C) dehydration of **35** to **21**, D) dehydration of **36** to **22**.

Selina-5,7(11)-diene (**20**) can arise from **I1** through 1,2-hydride shift to **I1a** and deprotonation (Scheme 7). This compound was first reported from olibanum oil, but only identified from its mass spectrum and GC retention time [90]. This structural assignment in the absence of a reference standard or at least literature data for **20** is likely erroneous. Compound (−)-**20** was later obtained by thermal degradation of (+)-maalian-5-ol (**33**) (Scheme 10A) and upon treatment of 4-*epi*-maaliol (**34**) with acid (Scheme 10B). Full ^1^H and ^13^C NMR data for **20** were reported [91]. Compound **21** can in theory be formed from **I1a** by 1,2-methyl group shift to **I1b** and deprotonation (Scheme 7). However, this compound was only obtained as synthetic material by dehydration of (−)-1(10)-valencen-7β-ol (**35**) (Scheme 10C) [92], but has not been isolated from natural sources. Compound **22** could be formed from **I1a** by Wagner–Meerwein rearrangement to **I1c** and deprotonation (Scheme 7). This hydrocarbon ([α]_D_^22^ = +26, *c* 0.06) has been obtained as a dehydration product of (−)-hinesol (**36**) (Scheme 10D), but has never been isolated from natural sources. ^1^H NMR data have been reported [92].

### Eudesmanes from **I2**

**Scheme 11:**
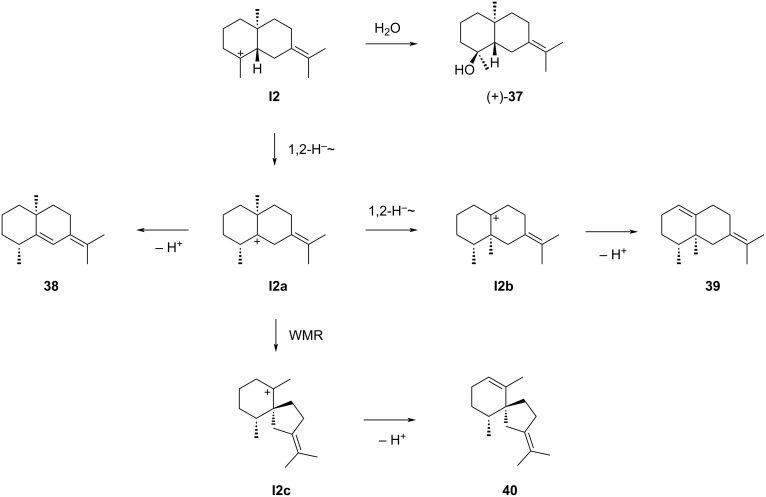
The sesquiterpenes derived from cation **I2**. WMR = Wagner–Meerwein rearrangement.

**Scheme 12:**
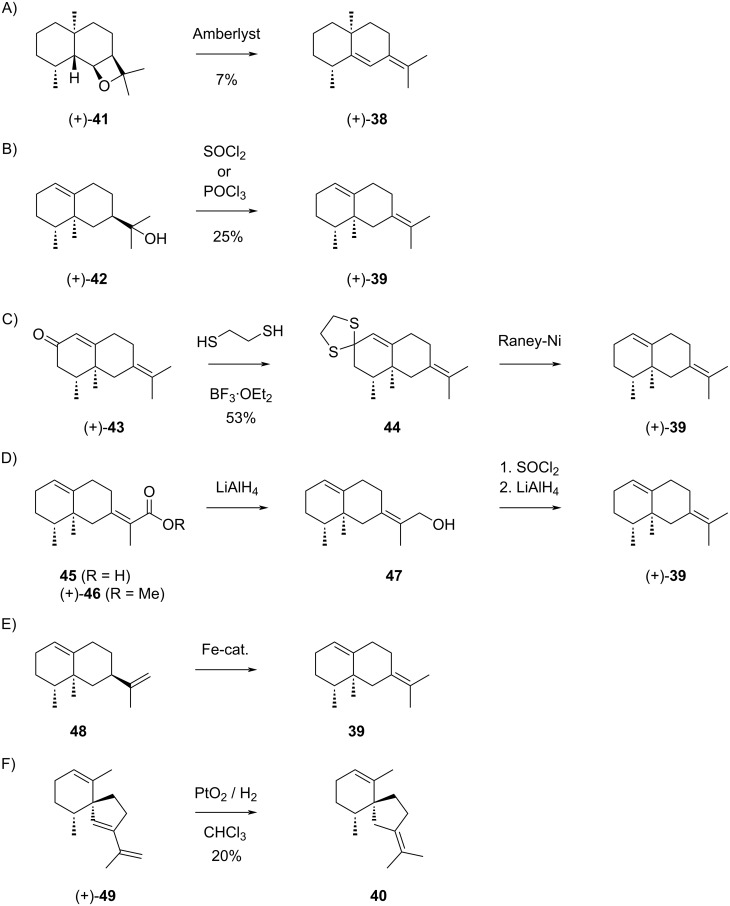
The sesquiterpenes derived from cation **I2**. A) Acid catalysed conversion of **41** into **38**, B) dehydration of **42** to **39**, C) chemical correlation of **43** with **39**, D) chemical correlation of **45** with **39** (no yields were given in the original report), E) isomerisation of **48** to **39** (product was not isolated), F) partial hydrogenation of **49** to **40**.

Much less is known about sesquiterpenes derived from cation **I2** (Scheme 11). The compounds described in the literature include (+)-juniper camphor (**37**) that can be formed by attack of water to **I2**. As mentioned above, this compound occurs in *Cinnamomum camphora* [86] and has later also been isolated from *Laggera pterodonta* ([α]_D_^24^ = +4, *c* 0.5, MeOH) [93]. Compound **38**, (+)-eudesma-5,7(11)-diene, could potentially arise from **I2** by 1,2-hydride shift to **I2a** and deprotonation, but has not been isolated from natural sources. This material was obtained by treatment of (+)-6,11-epoxyeudesmane (**41**) with acidic ion exchange resin (Scheme 12A) [94].

Also 4βH,5α-eremophila-1(10),7(11)-diene (**39**), biosynthetically accessible from **I2a** by 1,2-methyl shift to **I2b** and deprotonation (Scheme 11), is only known as a synthetic compound. This hydrocarbon has first been obtained by dehydration of (+)-valerianol (**42**) with SOCl_2_ or POCl_3_, yielding – besides the Hofmann product as main product (75%) – (+)-**39** (25%, [α]_D_^20^ = +167.5, neat) (Scheme 12B) [95]. After the first description of **39**, also (+)-α-vetivone (**43**) (Scheme 12C) [96–97] and isovalencenic acid (**45**) (Scheme 12D) [98] were correlated to this hydrocarbon. Recently, an iron catalyst has been developed that was applied in the isomerisation of valencene (**48**) to **39** (Scheme 12E) [99]. The biogenesis of **40** would be possible from **I2a** through Wagner–Meerwein rearrangement to **I2c** and deprotonation, but also this compound is not known as a natural product. This hydrocarbon has been obtained by partial hydrogenation of (+)-α-vetispirene (**49**) in a small scale reaction using PtO_2_ hydrate in CHCl_3_ as a catalyst (Scheme 12F). The amounts of isolated **40** (0.2 mg) were insufficient for a full spectroscopic characterisation [92].

### Eudesmanes from **I3**

**Scheme 13:**
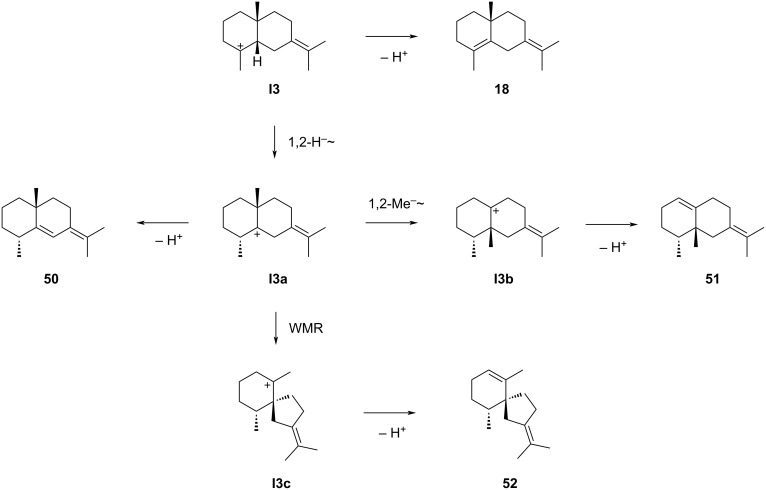
The sesquiterpenes derived from cation **I3**. WMR = Wagner–Meerwein rearrangement.

Also only a few compounds potentially arising from **I3** are known (Scheme 13). Compound **18** was already discussed above and can be formed by deprotonation from **I1** or **I3**. Cation **I1** seems to be the more likely precursor than **I3**, because **I1** is the intermediate towards structurally related natural products such as the widespread compounds **9** and **10** and a common biosynthesis of **18** through the same intermediate can be assumed (Scheme 7). A 1,2-hydride shift to **I3a** and deprotonation could give rise to **50**, a compound for which the situation in the literature is very confusing. There is no paper available describing the isolation and structure elucidation of a compound with the structure of **50**, and the first published paper that can be found under the CAS number of **50** (869998-21-0) does not mention this compound [100]. Several later reports claim the detection of “eudesma-5,7(11)-diene”, a name assigned to CAS number 869998-21-0, but neither a structure is shown nor a reference to previous work is given in these reports, leaving doubt about the stereostructure the authors of this work had in mind [101–103]. One recent report mentions the detection of “eudesma-5,7(11)-diene”, but again no structure is shown, and the structural assignment is based on a comparison of retention indices [104]. However, the deviation between measured and reference retention index is quite large (*I* = 1572 vs 1543), and the reference data originate from [103] in which the basis for structural assignment is unclear. Finally, one more paper assigned to CAS number 869998-21-0 mentions the detection of “eudesma-5,7(11)-diene”, but in this case the structure of **38** (Scheme 11) instead of **50** is shown, which based on a comparison of the measured to a database retention index may at least in terms of the relative configuration be a correct structural assignment [105]. Taken together, the confusing situation for **50** in the literature demonstrates impressively, how inaccurate data reporting can lead to unclear structural assignments and even error propagation, and shows the importance of structure elucidation by classical methods, i.e., isolation and compound characterisation by NMR spectroscopy and determination of optical rotation.

Compound **51** can be generated biosynthetically from **I3a** through 1,2-methyl migration to **I3b** and deprotonation. However, this hydrocarbon has not been isolated from natural sources and is only known as racemic synthetic material [106]. Similarly, **52** has only been described as a synthetic compound [107]. Its hypothetical biosynthesis is possible from **I3a** by Wagner–Meerwein rearrangement to **I3c** and deprotonation.

### Guaianes

**Scheme 14:**
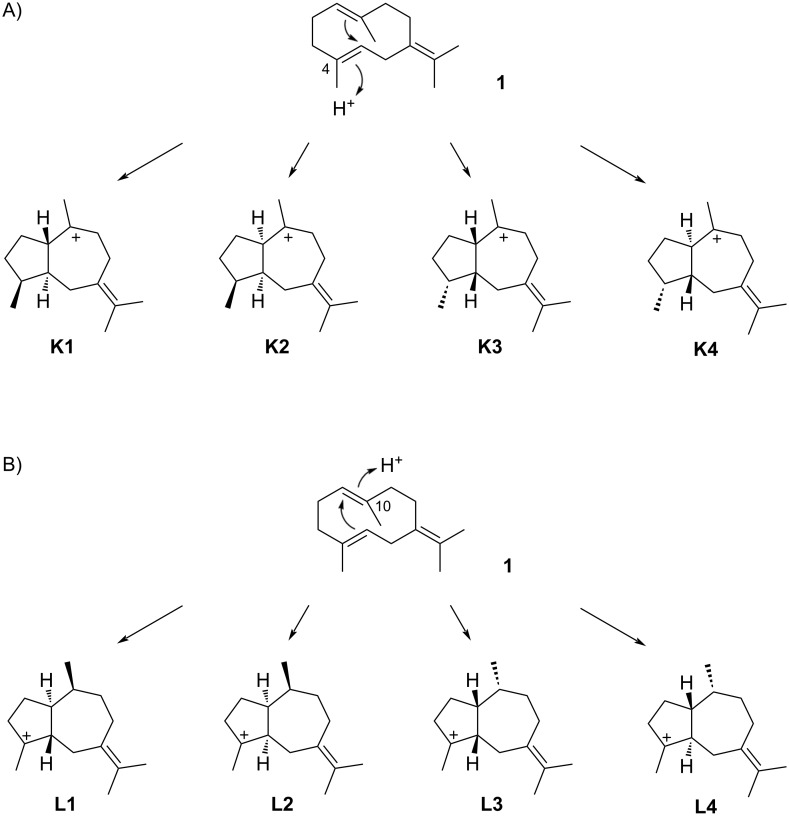
Cyclisation modes for **1** to the guaiane skeleton. A) The reprotonation of **1** at C-4 potentially leads to four stereoisomers of cation **K**, B) reprotonation at C-10 can result in four stereoisomers of **L**.

As discussed above, the cyclisation of **1** induced by reprotonation at C-4 to the eudesmane skeleton encounters obstacles because of the formation of secondary cations. Preferentially, reprotonation at C-4 leads to the guaiane skeleton since the formed cations are tertiary. Alternatively, reprotonation of **1** at C-10 can also induce the formation of the guaiane skeleton. Assuming *anti* addition to the C-4/C-5 double bond in **1**, only four cationic intermediates (**K1**–**K4**) can be generated by reprotonation at C-4 (Scheme 14A). Similarly, reprotonation of **1** at C-10 leads by *anti* addition to the C-1/C-10 double bond to four cationic intermediates, **L1**–**L4** (Scheme 14B).

**Scheme 15:**
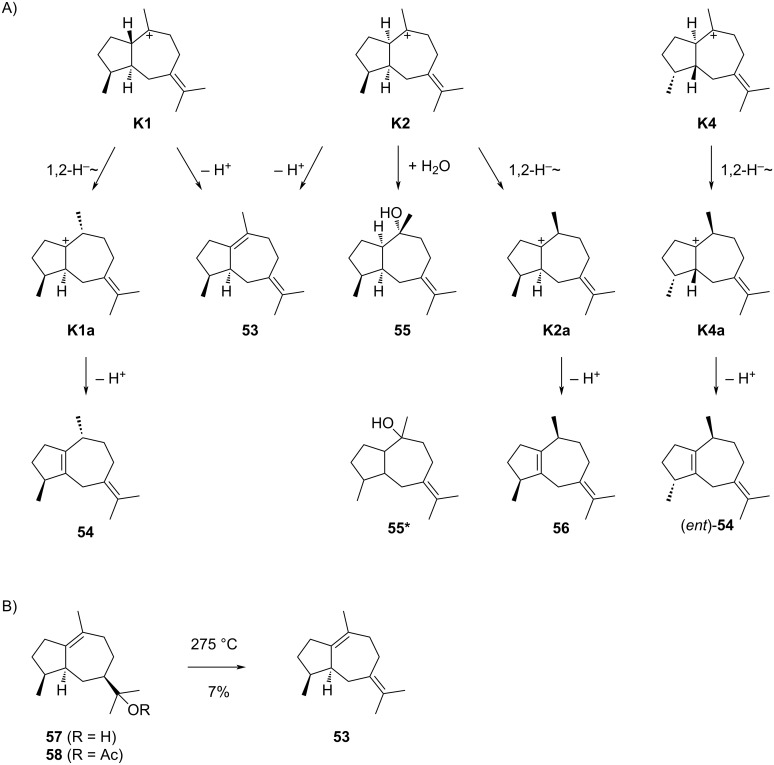
The sesquiterpenes derived from cations **K1**, **K2** and **K4**. A) Mechanisms of formation for compounds **53**–**56**, B) pyrolysis of **58** to **53**.

The guaiane sesquiterpenes derived from cationic intermediates **K1**, **K2** and **K4** are summarised in Scheme 15A, while no compounds are known whose formation could be explained from **K3**. β-Bulnesene (**53**), a product by the deprotonation of **K1** or **K2**, was first isolated from the guaiac wood oil of *Bulnesia sarmientoi* [108] and later also observed in *Pogostemon cablin* [109]. Bulnesol (**57**), a compound of known absolute configuration [110] that occurs in the same essential oil [108], has been converted through pyrolysis of its acetate **58** into **53** (Scheme 15B) [111], securing the relative configuration. This work did not comment on the question of absolute configuration, but assuming a common biosynthesis of **53** and **57** analogous absolute configurations for these compounds are likely. Despite several reported syntheses of (*rac*)-**53** [112–116], no enantioselective synthesis is available. Full ^1^H and ^13^C NMR data of **53** (including 14 carbon signals) have been published [113].

**Scheme 16:**
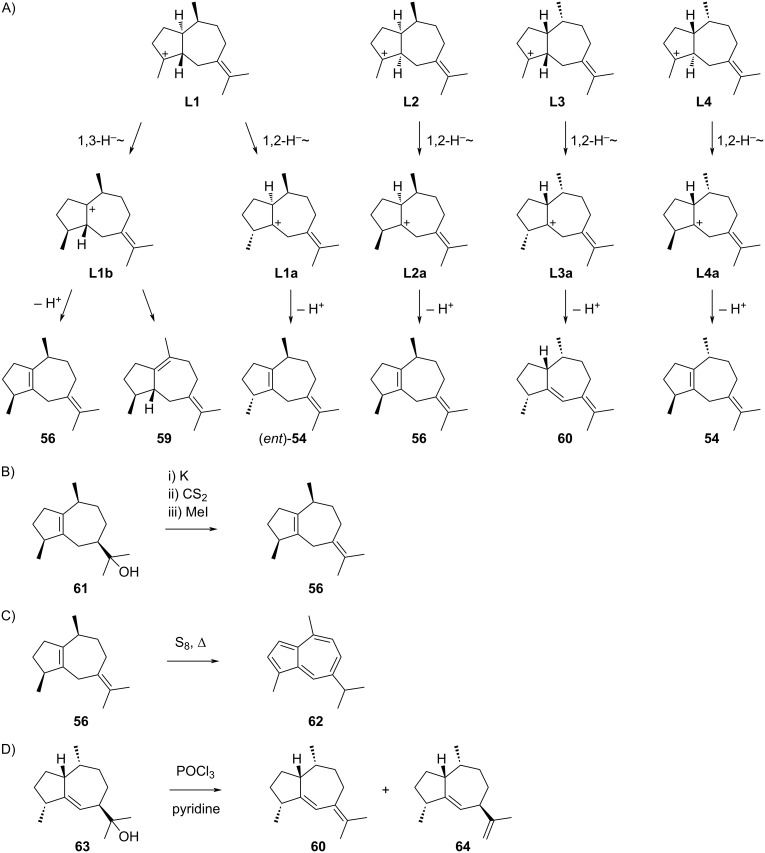
The sesquiterpenes derived from cations **L1–L4**. A) Mechanisms of formation for compounds **54**, **56**, **59** and **60**, B) dehydration of **61** to **56**, C) oxidation of **56** to **62**, D) dehydration of **63** to **60** and **64** (no yields were given in the original reports for the synthetic transformations shown in this Scheme).

The guaiane sesquiterpenes that are potentially derived from cationic intermediates **L1**–**L4** are summarised in Scheme 16A. *trans*-β-Guaiene (**54**) can either be generated from **K1** undergoing a 1,2-hydride shift to **K1a** followed by deprotonation (Scheme 15A), or from **L4** through a similar sequence of steps (Scheme 16A). Its enantiomer *ent*-**54** could analogously arise from **K4** or **L1**. The first detection of this compound was claimed from *Aframomum alboviolaceum*, but this study did not report on the isolation and structure elucidation [117]. Rather the identification was only based on GC–MS data, without a reference to a previous identification through rigorous structure elucidation. Conclusively, this compound has not been described thoroughly and its identification is doubtful. Information about the mass spectrum and Kovats retention index have been added to data bases such as the NIST Chemistry Webbook [118], which promoted the ambiguous detection of **54** in many other species, as described in more than 300 papers to date.

Compound **55** can be formed from **K2** through capture with water. A compound with the same planar structure of 55* named guai-7(11)-en-10-ol has been reported from *Zanthoxylum syncarpum* with fully assigned ^1^H and ^13^C NMR data, but unresolved relative and absolute configuration [119]. For unclear reason, this compound has been assigned to CAS number 461691-86-1, a molecule for which the relative and absolute configuration are shown. No other reports for this compound are available.

β-Guaiene (**56**) is a well described compound that can biosynthetically arise from **K2** by a 1,2-hydride shift to **K2a** and deprotonation (Scheme 15A), or alternatively from **L2** through similar reactions, or from **L1** by 1,3-hydride shift to **L1b** and deprotonation (Scheme 16A). DFT calculations have shown that such 1,3-hydride shifts are only possible for *trans*-fused guaiane systems [120]. Without detailed knowledge about the structure, β-guaiene (**56**) was first obtained from guaiol (**61**) by Wallach in 1894 [121] and again prepared by Gandurin in 1908 by elimination of the instable methyl xanthogenate (Scheme 16B) [122], followed by an isolation from *Acorus calamus* ([α]_D_^20^ = +13) by Šorm and co-workers [123]. It is well known that **56** can easily be dehydrogenated, e.g., by heating with sulphur, to the blue azulene derivative **62** (Scheme 16C) [121–122,124–126], but the structure elucidation of this compound was only completed in 1936 [127]. Based on a comparison of IR spectra of natural terpenes, their hydrogenation and dehydrogenation products, the correct planar structure of **56** was concluded by Pliva and Šorm [128]. After the absolute configuration of **61** was solved [129], the full stereostructure of **56** became known. No total synthesis and no NMR data are available for **56**. β-Guaiene is one of the main constituents of the essential oil from *Achillea millefolium* that shows inhibitory activity against *Babesia canis*, a parasite transmitted by ticks that infects blood cells [130].

Compound **59** is accessible by deprotonation of **L1b**, but only known as synthetic racemic material [113–116]. Compound **60** can be produced by cationic intermediate **L3** through 1,2-hydride shift to **L3a** and deprotonation (Scheme 16A). However, this compound itself is not known as a natural product, but has been obtained together with γ-gurjunene (**64**) from guai-11-en-5-ol (**63**), a natural product isolated from gurjun wood oil, by elimination (Scheme 16D) [131].

## Conclusion

As summarised in this review, the biosynthesis of many sesquiterpene hydrocarbons and alcohols exhibiting the eudesmane or guaiane skeleton can be explained from the neutral intermediate germacrene B, although not all compounds known to literature have been isolated from natural sources; some compounds are only known as synthetic materials. Compared to the known compounds arising from germacrene A or hedycaryol through similar reactions as discussed here [12–13], however, the number of terpenes derived from germacrene B is much lower. In this article we have explained the rationale for the structure elucidation including relative and, if known, absolute configurations. Through a detailed analysis of the available information it also turned out that some of the assigned structures are doubtful. The importance of rigorous structure elucidation, historically usually performed by chemical correlations and today preferentially done by NMR spectroscopy or X-ray analysis, is clearly evident from the fact that wrongly reported structures or structures assigned without any comprehensible basis lead to error propagations and highly confusing situations in the literature. Today many reports are only based on tentative GC–MS assignments, often even without comparison to authentic standards, which results in a lot of information of questionable relevance. The large number of such papers published today makes it more and more difficult to find the relevant information in the literature. With this work we hope to help the interested reader to have an easier access to the knowledge about sesquiterpenes derived from germacrene B.
